# A DArT marker genetic map of perennial ryegrass (*Lolium perenne* L.) integrated with detailed comparative mapping information; comparison with existing DArT marker genetic maps of *Lolium perenne*, *L. multiflorum* and *Festuca pratensis*

**DOI:** 10.1186/1471-2164-14-437

**Published:** 2013-07-03

**Authors:** Julie King, Ann Thomas, Caron James, Ian King, Ian Armstead

**Affiliations:** 1 School of Biosciences, University of Nottingham, Sutton Bonington Campus, Loughborough, Leicestershire LE12 5RDs, UK; 2 Institute of Biological, Environmental and Rural Sciences, Gogerddan Campus, Aberystwyth, Ceredigion SY23 3EE, UK

**Keywords:** DArT Markers, Genetic Maps, *Lolium*, *Festuca*, Comparative Genomics

## Abstract

**Background:**

Ryegrasses and fescues (genera, *Lolium* and *Festuca*) are species of forage and turf grasses which are used widely in agricultural and amenity situations. They are classified within the sub-family Pooideae and so are closely related to *Brachypodium distachyon*, wheat, barley, rye and oats. Recently, a DArT array has been developed which can be used in generating marker and mapping information for ryegrasses and fescues. This represents a potential common marker set for ryegrass and fescue researchers which can be linked through to comparative genomic information for the grasses.

**Results:**

A F2 perennial ryegrass genetic map was developed consisting of 7 linkage groups defined by 1316 markers and deriving a total map length of 683 cM. The marker set included 866 DArT and 315 gene sequence-based markers. Comparison with previous DArT mapping studies in perennial and Italian ryegrass (*L. multiflorum*) identified 87 and 105 DArT markers in common, respectively, of which 94% and 87% mapped to homoeologous linkage groups. A similar comparison with meadow fescue (*F. pratensis*) identified only 28 DArT markers in common, of which c. 50% mapped to non-homoelogous linkage groups. In *L. perenne*, the genetic distance spanned by the DArT markers encompassed the majority of the regions that could be described in terms of comparative genomic relationships with rice, *Brachypodium distachyon*, and *Sorghum bicolor*.

**Conclusions:**

DArT markers are likely to be a useful common marker resource for ryegrasses and fescues, though the success in aligning different populations through the mapping of common markers will be influenced by degrees of population interrelatedness. The detailed mapping of DArT and gene-based markers in this study potentially allows comparative relationships to be derived in future mapping populations characterised using solely DArT markers.

## Background

Perennial ryegrass (*Lolium perenne*) is the most important forage grass of N. Europe and is also grown widely in temperate regions worldwide. It is a member of a group of inter-fertile, largely outbreeding, related species which encompass a number of ryegrasses and fescues (*Lolium* and *Festuca* spp.) including, Italian ryegrass (*L. multiflorum*) and meadow and tall fescue (*F. pratensis* and *F. arundinacea*). Because of their economic significance and evolutionary relationships, there has been interest in characterising these grasses in molecular terms both as individual species and as ancestral and recent hybrids.

Genetic maps have been established for ryegrasses and fescues largely as a consequence of the desire to understand the genetic architecture of both QTL and single genes which confer agriculturally important traits. Marker-based maps have progressed from linkage groups developed largely from restriction fragment-length polymorphisms (RFLPs) [1-5] to those developed from combinations of random amplified polymorphic DNA (RAPDs), amplified fragment length polymorphisms (AFLPs) and microsatellites/simple sequence repeats (SSRs) [6-13]. While these have delivered their immediate aims, the lack of a comprehensive common set of reference markers has limited the number of opportunities to align these maps and, so, the associated trait data produced for different studies. Recognition of this for ryegrasses and fescues led to the manufacture of a Diversity Array Technology (DArT) array constructed from 40 ryegrass and fescue accessions [14] and a related open access database [15]. The hope was that this would encourage the use of a common marker set and so enable more efficient exploitation of the available genetic data for ryegrasses and fescues. This, in turn, would contribute towards both increasing our understanding of the genetics and biology of these grasses and the development of protocols for molecular breeding. The publication of perennial and Italian ryegrass and meadow fescue genetic maps and analyses of *Lolium x Festuca* hybrids [16,17] integrating DArT markers indicates that this process is underway.

Ryegrasses are classified in the Pooideae subfamily within the Poaceae, along with wheat, barley, rye and oats, as well as the model monocot *Brachypodium distachyon*. Thus, ryegrasses and fescues are part of a group of closely related important model and crop species. Within the same clade (BEP), but in a different subfamily (Erhartoideae) is the other major monocot model and crop, rice. More distantly related grasses are classified in the PACMAD clade, which includes many of the tropical C4 cereals and grasses such as maize, *Sorghum bicolor*, pearl millet, sugar cane and *Miscanthus* spp [18]. For three of these grasses in particular, rice [19], *B. distachyon*[20], and *S. bicolor*[21]*,* comprehensive genome assemblies and pseudomolecules have now been released; these enable whole genome sequence comparisons to be made between these species. Additionally, this allows for the alignment of genetic maps constructed from sequence-based markers to be made with these whole genome assemblies, so identifying conserved marker/sequence orders between species. From these orders, macrosyntenic relationships can be imputed in the absence of comprehensive sequence-based assemblies for ryegrasses and fescues. Thus, in ryegrass, for which there is currently limited publicly available sequence information, it has been possible to use its conserved syntenic relationships with the sequenced model genome of rice [22,23] to suggest candidate genes underlying QTL [13,24,25].

In this report, we will describe the construction of a genetic linkage map in ryegrass consisting predominantly of DArT and gene-based markers. This allows for the DArT markers to be assigned to putative comparative genomic regions within this framework.

## Methods

### Plant material

The *L. perenne (Lp)* F2 mapping population (Lp1) used was based on a cross between inbred genotypes derived from the varieties Perma and Aurora. The development and use of this mapping population has been described previously [26].

### Marker assays

Previous studies have reported the incorporation of RFLP, AFLP, isozyme, SSR and gene-based SNP markers into this F2 linkage map [2,13,22,26,27]. New markers developed from the present study were based on DArT technology.

For the DArT markers, DNA was extracted from 94 genotypes of the F2 family using the QIAGEN DNeasy 96 Plant Kit (QIAGEN, Crawley, UK). These samples were processed on the high-density Lolium/Festuca DArT arrays [14] at Diversity Arrays Technology Pty Ltd, Canberra, Australia [28] and segregating markers identified. All DArT markers were given the prefix D (substituting ‘loPt-‘) followed by the unique six number identifier. For the gene-based SNP markers used for placing DArT markers in a comparative genomics context, the development rationale, primer and marker sequences and assays have been described previously [22]. Briefly, putative intra-exon PCR primers were designed based upon gene models developed as part of the rice (*Oryza sativa; Os*) genome sequencing programme [29] using gene models which spanned all 12 pseudomolecules of the rice genome. Using these primers, amplicons were derived from a DNA template pool consisting of equal quantities of genomic DNA from 96 individuals of the Lp1 mapping family (effectively recreating the genomic constitution of the original F1). Potential SNPs were identified by visual inspection of the chromatogram files and detected in the mapping family using protocols based on KASP technology [30]. KASP assays were developed and implemented by LGC Genomics Ltd., Herts., UK.

### Genetic mapping

In order to minimise the number of missing data points the data was processed in two ways after scoring. Firstly, the family size was reduced to 86 genotypes which had been used for the generation of the majority of marker types (i.e., DArT, SNP and all the previously scored markers). Secondly, markers which gave identical segregation patterns, except for missing data, were all mapped as a single marker with missing genotype scores derived from the consensus. Genetic mapping on these datasets was carried out using JoinMap 3.0 [31] using the default conditions. Markers which could not be incorporated in rounds one or two (see Joinmap 3.0) were omitted from the final maps, but their likeliest positions reported in Additional file 1.

### Comparative mapping

As described by King et al. [22], where sequences had been generated for mapped markers (e.g. the SNP markers) or were already published (many RFLP markers), these sequences were aligned with complete pseudomolecule (PM) assemblies for *Os *[29]; (v6.1/all_con), *Brachypodium distachyon* (*Bd*) and *Sorghum bicolor* (*Sb*) [32] (Bdistachyon_114.fa and Sbicolor_79.fa) using BLAST-generated sequence alignments with a cutoff threshold of e = <1e-005; see (Additional file 2). In each case, only the most significant BLAST alignment was used to assign putative orthology between the *Lp* marker sequences and the PM sequences for *Os*, *Bd* and *Sb*. The comparative relationships were described as plots of the genetic positions (cM) of sequenced markers on the *Lp* linkage groups (LG) against the physical positions (bp) of the putative orthologues on the *Os*, *Bd* and *Sb* PMs. In all cases, the genetic and physical positions were expressed as percentages of the total genetic distance of the LG or the complete PM length.

## Results

### Genetic mapping

**Figure 1:**
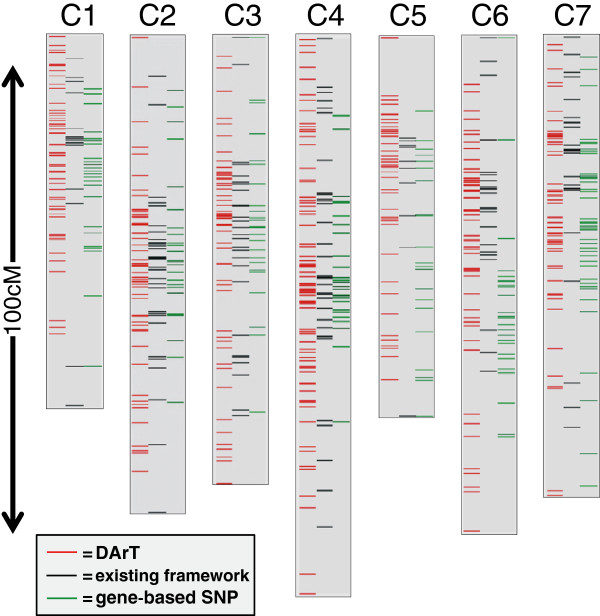
***L. perenne *genetic map.** Genetic map of the *L. perenne* F2 mapping population illustrating the relative distributions of marker types over the 7 linkage groups (C1-C7). For each linkage group, column 1 contains the DArT marker positions, column 2 framework marker positions excluding gene-based SNPs and column 3 the gene-based SNP marker positions.

Genetic mapping using JoinMap3.0 incorporated the new DArT data into the existing framework maps for this F2 population giving an overall map length of 683 cM defined by 1316 markers; an additional 66 DArT, 3 SNP, 3 SSR and a single AFLP marker could be assigned ‘best-fit’ positions (Additional file 1). The average interval between segregating markers was 0.92 cM, with the largest interval being 13.4 cM. Significant regions of distorted segregation were detected on LGs 5 and 7, as has previously been reported for this family [27]. The overall distribution of the markers was typical for linkage groups constructed for plant species with moderate to large sized genomes, with marked clustering (reduced recombination) around the putative centromeric regions and higher levels of recombination towards the ends of the linkage groups. The distribution of the different marker types is illustrated in Figure 1 and demonstrates the comprehensive genome sampling of the DArT markers relative to the gene-based SNP and other marker types, moderated by relative differences in recombination frequencies along the length of the LGs. Because of the low recombination frequencies in certain regions and the restricted family size, 771 markers (59%) showed no recombination with at least one other marker (Additional file 1).

#### DArT marker assays

Segregation data for a total of 1150 DArT markers was recorded, of which 942 could be associated with one or more markers in the existing LGs for the F2 family with a LOD ≥3. Of these 942, 867 could be assigned direct positions in the linkage maps and a further 66 ‘best-fit’ positions (Additional file 1). A comparison of maps produced with and without the inclusion of the DArT markers indicated no significant changes to marker orders (data not shown; compare with King et al. 2012 [22]). The overall total map length increased from 597 cM to 683 cM, this increase being due to the identification of new terminal markers, particularly on LGs 5, 6 and 7, rather than map expansion within the existing framework (Table 1). The DArT markers were fairly evenly distributed between the LGs except for LG4 to which 21% of the total number of DArT markers were assigned (mean per linkage group = 14.3%; Table 1). LG4 is also the longest linkage group (18% of the total genetic distance) and so the comparatively large number of DArT markers which map to LG4 may just be a reflection of this in combination with background variation. A similar pattern of results has previously been reported for *Lp* and *Lm *[16,33]. However, it is possible that potential DArT markers (i.e.’ those selected for the array) may not have an even distribution across the genome.

**Table 1:** The effect of DArT marker inclusion on the overall length of the linkage groups

	**No. DArT markers**	**% total mapped DArt markers**	**cM excluding DArT markers**	**cM including DArT markers**	**cM increase**
C1	138	15.9	77.2	78.5	1.3
C2	124	14.3	94.8	101.3	6.5
C3	99	11.4	90.1	96.0	5.9
C4	182	21.0	115.9	121.1	5.1
C5	121	14.0	62.3	82.1	19.7
C6	108	12.5	73.2	106.6	33.4
C7	95	11.0	83.8	97.5	13.7
Total	867	100	597.4	683.1	85.6

### Comparative mapping

**Figure 2:**
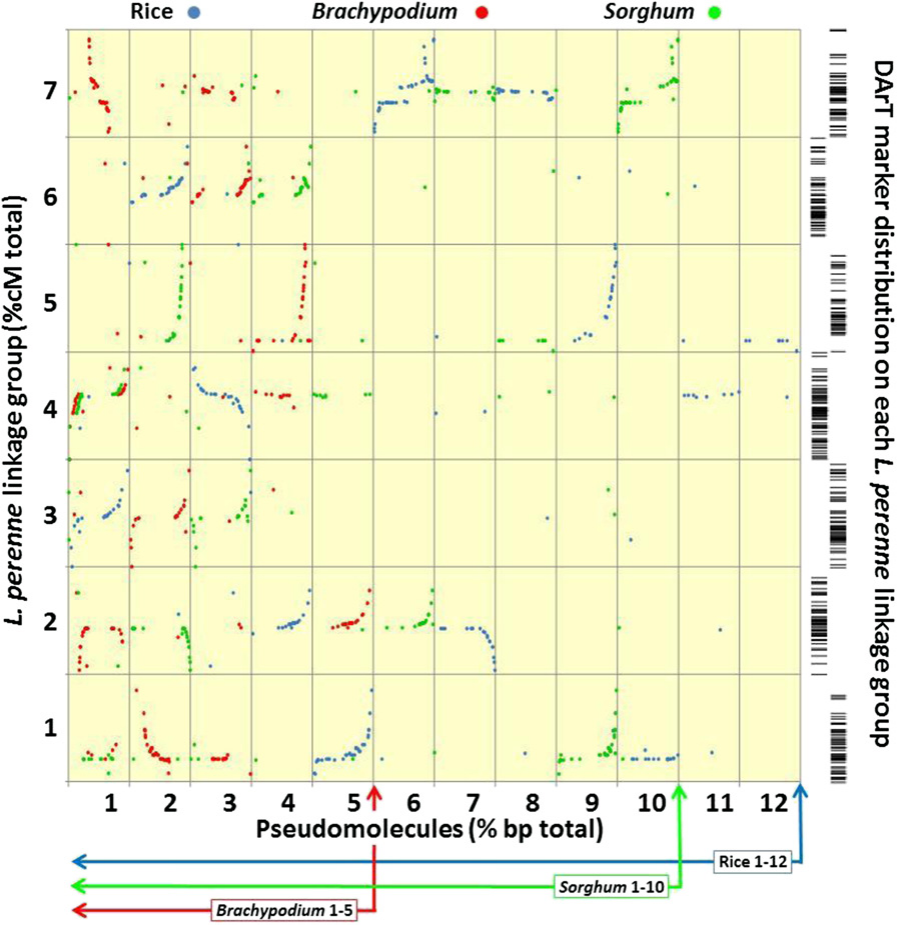
**Comparative genomic relationships between *L. perenne, B. distachyon*, *S. bicolor *and rice relative to DArT marker distribution.** Scatter plot illustrating the conserved syntenic relationships between the relative position of gene-based markers on the *L. perenne* linkage groups (Lp1 study), the physical position of their most probable sequence alignments on the *B. distachyon*, *S. bicolor* and rice pseudomolecules and the genetic distance spanned by the DArT markers mapped to each *L. perenne* linkage group. The position of the *S. bicolor* markers from pseudomolecule 1 which align with genetic markers which map to *L. perenne* linkage group 4 are offset slightly to avoid direct overlap with the *B. distachyon* alignments to the same linkage group. Figure adapted from King et al. [22].

A total of 315 gene-based markers mapped to the Lp1 family were used for establishing alignments with *Os*, *Bd* and *Sb* (Figure 2). These consisted of the 271 SNP markers, 37 RFLPs and 7 sequence tagged sites (STS). The mapping of these SNP markers in the absence of the DArT markers has been described previously [22] and the results are highly consistent with that study in terms of map orders and derived conserved syntenic relationships and also consistent with other *Lolium* and *Festuca* spp. studies in terms of comparative genomics [2,4,5,13,23,27,34].

## Discussion

While molecular genetic technologies have transformed our understanding of plant genome compositions and their interrelationships, they have yet to have a major impact on the plant breeding of many crops. This is largely due to the perceived cost/benefit ratio of applying these molecular technologies in different biological and commercial contexts, *i.e.*, as influenced by the breeding systems and genetic complexities of different commercial plant varieties and the immediate economic value of individual crops. Ryegrasses are particularly challenging for the molecular breeder in that: a) they are, in the main, obligate outbreeders and so highly heterozygous; b) marketed varieties are frequently synthetic populations which are, genetically speaking, very distant from the tractable inbred lines predominant for barley, wheat and the F1 hybrids of maize; c) the main commercial value of ryegrass breeding is in the products of grassland agriculture rather than in the seed sales *per se*. As a consequence, molecular breeding of ryegrasses is still very much in development. As a contribution to this, the major purpose of this report is to add to the public body of knowledge on the genetic distribution of available marker sets for ryegrass and the relationship of these to established molecular genetic platforms as represented by the sequenced plant genomes of *Os*, *Bd* and *Sb*. This should allow the molecular ryegrass breeder to obtain extra leverage from these genetic and genomic resources in terms of comparative QTL analysis, gene prediction and consequent marker development.

Currently, there are few ryegrass and fescue studies that have allowed for detailed genome-wide comparisons to be made between different genetic maps; where present, linkages between different studies are dependent on the use of either a limited number of ‘historic’ RFLP and/or SSR markers [2,10,13]. The development of a high-density DArT marker array for ryegrasses and fescues now means that a large, common marker set can be applied to different ryegrass and fescue populations which can result in high density genetic maps and measurements of population interrelatedness. The major disadvantage to using DArT markers (as with AFLPs) in many population types is their genetic dominance, *i.e.*, a DArT marker (allele) scored as present in a genotype could be either in the homozygous or heterozygous form. This has consequences in terms of predictive ability for linkages, allele frequencies and for marker trait associations. However, this is balanced by the ability of DArT markers to ‘capture’ the genetic space and it is sometimes possible to infer the dosage of a particular allele from linked co-dominant markers.

In this study we have presented a genetic map which places DArT markers in a detailed comparative genomic context. In order to incorporate as many markers of different types as possible, a reduced population size was employed with marker redundancy kept as low as possible. This minimised the number of missing data points which simplified the process of marker ordering on the individual linkage groups using JoinMap3.0. The consequence of this is that we were less likely to discover rarer recombination events that may have broken up some of the larger non-recombinant blocks of markers (Additional file 1). However, the uneven patterns of recombination we observed are not, primarily, artefacts of this experimental design and such patterns are consistently reported in ryegrass mapping studies with different population sizes [1,2,8,12,16,33]. In summary, while this mapping approach may not describe all the recombination present, we hope that by combining the maximum number of DArT and gene-based markers in a single map and demonstrating that the DArT markers encompass the majority of the ‘comparative genome’ (Figure 1 and 2) it will enable inferences to be made about putative comparative relationships in ryegrass and fescue mapping studies based mostly (or entirely) on DArT markers. This may be particularly useful in population surveys which are not based on bi-parental mapping family designs (for detailed alignments between *L. perenne* and wheat and barley, see [22]).

**Figure 3:**
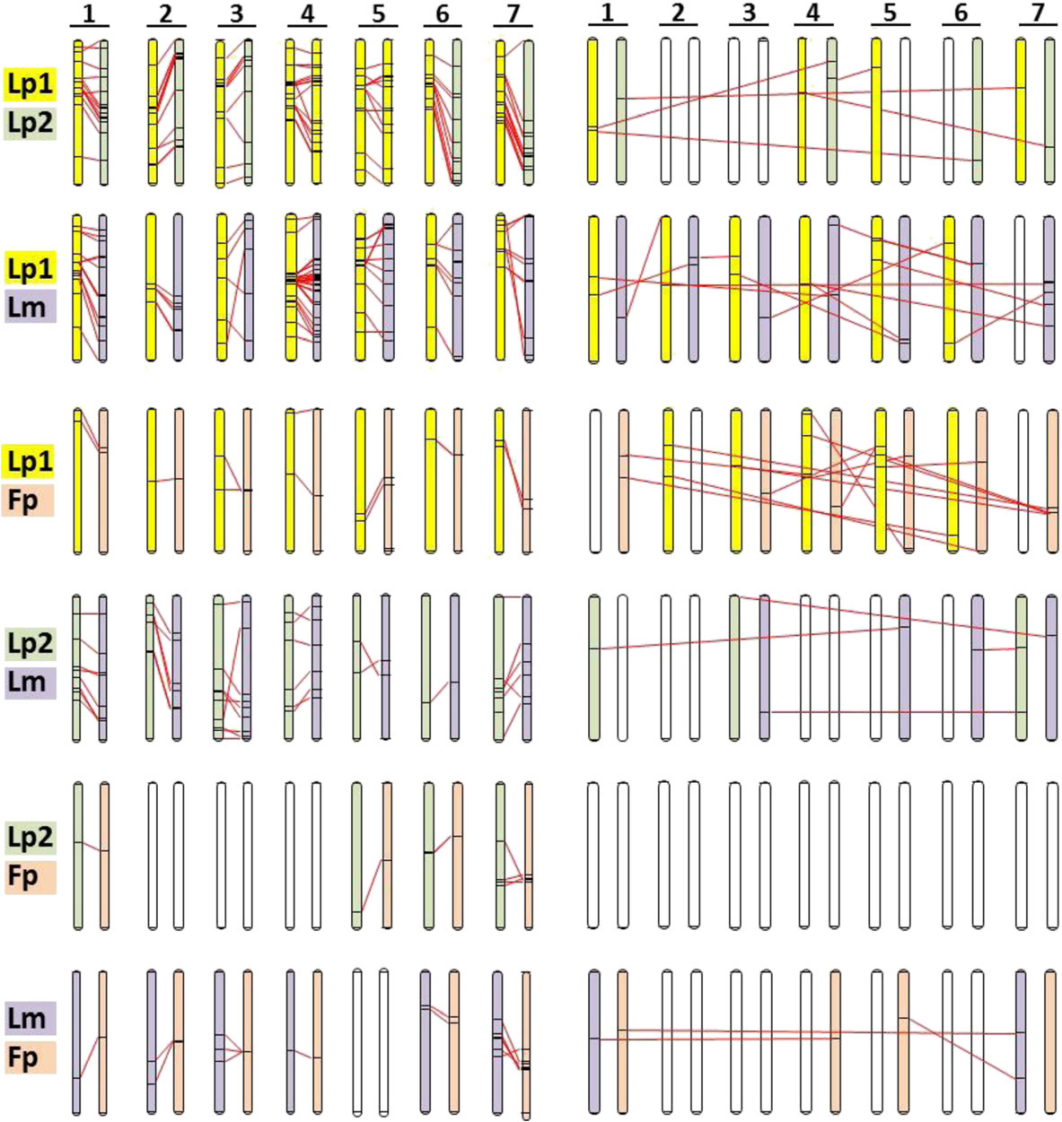
**DArT marker alignments between mapping studies in *L. perenne*, *L. multiflorum *and *F. pratensis*.** A comparison of genetic positions of DArT markers common to *L. perenne* (Lp1, this study, yellow; Lp2, green [33]) *L. multiflorum (*Lm*, purple) and F. pratensis* (Fp, orange) [16] mapping populations. The 7 pairs of linkage groups on the left illustrate the relative positions of common markers which map to the same linkage group and those on the right, the relative positions of common markers which map to different linkage groups. The genetic position of each marker is expressed as a % of the total length (cM) of each linkage group.

Tomaszewski et al. [33] and Bartos et al. [16] recently published studies which integrated DArT markers into linkage maps developed for *L perenne.* (Lp2) *L. multiflorum* (Lm) and *F. pratensis* (Fp) mapping populations. The total number of DArT markers mapped in the different populations varied from 867 to 149 (Table 2) and comparisons of the Lp1, Lp2 and Lm *Lolium* populations identified between 80 and 105 markers in common (on a pairwise comparison basis) of which >85% map to similar positions on homologous and homoeologous linkage groups (Figure 3; Additional file 3). Comparing the Lp1 and Lp2 populations, of the 297 possible shared loci (*i.e.* the smaller number of DArT markers mapped in either population) c. 28% were putatively allelic (*i.e.*, the same marker designation mapping to a similar position on the homologous/homoeologous linkage group); equivalent figures for the Lp1/Lm and Lp2/Lm comparisons were 17% and 13%, respectively and for the Lp1/Fp, Lp2/Fp and Lm/Fp comparisons, 9%, 5% and 11%, respectively. Thus, as would be expected, the greater the genetic distance between two genotypes or populations, the fewer the number of DArT markers that they had in common (a similar low number of common markers between the Lp1 population and a different Fp genotype has also been observed by the authors; unpublished).

**Table 2:** **Pairwise comparisons of DArT marker distributions between the *L. perenne*, *L. multiflorum *and *F. pratensis *mapping studies**

	**Lp1**	**Lp2**	**Lm**	**Fp**
Lp1	**867**	82	91	14
Lp2	*5*	**297**	38	8
Lm	*14*	*4*	**530**	17
Fp	*14*	*0*	*3*	**149**

Bold text diagonal - the number of DArT markers mapped independently in each of the four studies. Above the diagonal, plain text - the number of DArT markers common between studies and mapping to the same linkage group. Below the diagonal, italic text - the number of DArT markers common between studies and mapping to different linkage groups.

A number of DArT markers with the same identifiers were, apparently, non-allelic in different populations (Table 2 and Figure 3; Additional file 3). While it is possible that some of these ‘discrepancies’ result from genuine genome rearrangements, it is likely that many result from cross-hybridisation of similar sequences on the arrays. For the Lp1/Lp2 comparison, 6% (n = 87) of the DArT markers were non-allelic; for the Lp1/Lm and Lp2/Lm comparisons 13% (n = 105) and 10% (n = 42), respectively, were non-allelic. For the comparisons between Lp1, Lp2, Lm and Fp the equivalent figures were 50% (n = 28) 0% (n = 8) and 15% (n = 20), respectively. Clearly, the low numbers of common markers in these latter comparisons do not allow any firm conclusions to be drawn, but the Lp1/Fp results would suggest that non-allelic cross hybridisation is likely to complicate direct map comparisons in wider crosses. As more studies using the DArT marker array are reported, it will be interesting to see if a core set of ‘consistent’ markers emerges for mapping both within and between different populations of ryegrasses and fescues.

## Conclusion

This study describes an integration of 866 DArT markers into a comprehensive map of ryegrass which can be aligned with *B. distachyon*, rice and *S. bicolor*. In so doing, it is hoped that this will be useful resource for guiding the alignments of different ryegrass mapping and population studies, in order to maximise mutual transfers of information. It is likely that, in the near future, the sequencing and resequencing of the ryegrass genome will precipitate the development of economic genotyping-by-sequencing approaches which will be able to characterise allelic variation at far greater depth. However, until that is achieved common marker sets and comparative genomics are likely to guide the way forward for molecular plant breeding of ryegrasses.

## Competing interests

The authors declare that they have no competing interests.

## Authors’ contributions

JK, IK and IA conceived and designed the experiments; JK, AT, CJ and IA carried out the molecular genetic analyses; IA drafted the manuscript, JK, IK and IA compiled the manuscript. All authors read and approved the final manuscript.

## Supplementary Material

Additional file 1 **Genetic map of the Lp1 *L.* perenne F2 mapping population.** Click here for file

Additional file 2 BLAST results summary for gene-based markers. Click here for file

Additional file 3 Details of DArT markers mapped in common between the different mapping populations. Click here for file
